# Gene expression profiling of *Trypanosoma cruzi* in the presence of heme points to glycosomal metabolic adaptation of epimastigotes inside the vector

**DOI:** 10.1371/journal.pntd.0007945

**Published:** 2020-01-02

**Authors:** Marcia C. Paes, Francis M. S. Saraiva, Natália P. Nogueira, Carolina S. D. Vieira, Felipe A. Dias, Ana Rossini, Vitor Lima Coelho, Attilio Pane, Fei Sang, Marcos Alcocer

**Affiliations:** 1 Laboratório de Interação Tripanossomatídeos e Vetores—Departamento de Bioquímica, IBRAG–UERJ–Rio de Janeiro, Brazil; 2 Instituto Nacional de Ciência e Tecnologia—Entomologia Molecular (INCT-EM)–Brazil; 3 Laboratório de Bioquímica de Artrópodes Hematófagos, Instituto de Bioquímica Médica, Universidade Federal do Rio de Janeiro, Rio de Janeiro, Brazil; 4 Laboratório de Toxicologia e Biologia Molecular, Departamento de Bioquímica, IBRAG- UERJ- Rio de Janeiro, Brazil; 5 Instituto de Ciências Biomédicas, Universidade Federal do Rio de Janeiro, Rio de Janeiro, Brazil; 6 Deep Seq, School of Life Sciences, University of Nottingham, Nottingham, United Kingdom; 7 School of Biosciences, University of Nottingham, United Kingdom; University of Texas at El Paso, UNITED STATES

## Abstract

Chagas disease, also known as American trypanosomiasis, is a potentially life-threatening illness caused by the protozoan parasite, *Trypanosoma cruzi*, and is transmitted by triatomine insects during its blood meal. Proliferative epimastigotes forms thrive inside the insects in the presence of heme (iron protoporphyrin IX), an abundant product of blood digestion, however little is known about the metabolic outcome of this signaling molecule in the parasite. Trypanosomatids exhibit unusual gene transcription employing a polycistronic transcription mechanism through trans-splicing that regulates its life cycle. Using the Deep Seq transcriptome sequencing we characterized the heme induced transcriptome of epimastigotes and determined that most of the upregulated genes were related to glucose metabolism inside the glycosomes. These results were supported by the upregulation of glycosomal isoforms of PEPCK and fumarate reductase of heme-treated parasites, implying that the fermentation process was favored. Moreover, the downregulation of mitochondrial gene enzymes in the presence of heme also supported the hypothesis that heme shifts the parasite glycosomal glucose metabolism towards aerobic fermentation. These results are examples of the environmental metabolic plasticity inside the vector supporting ATP production, promoting epimastigotes proliferation and survival.

## Author summary

Chagas disease, also known as American trypanosomiasis, is a potentially life-threatening illness caused by the protozoan parasite, *Trypanosoma cruzi*, and is transmitted by triatomine insects during its blood meal. Proliferative epimastigotes forms thrive inside the insects in the presence of heme (iron protoporphyrin IX), an abundant product of blood digestion. This parasite, as well as other trypanosomatids exhibit unusual gene transcription employing a polycistronic transcription mechanisms through trans-splicing that regulates its life cycle. Using the Deep Seq transcriptome sequencing we characterized the heme induced transcriptome of epimastigotes and determined that most of the upregulated genes were related to glucose metabolism inside the glycosomes. This was supported by the upregulation of glycosomal isoforms of PEPCK and fumarate reductase of heme-treated parasites, implying that the fermentation process was favored. Moreover, the downregulation of mitochondrial enzymes in the presence of heme also supported the hypothesis that heme shifts the parasite glycosomal glucose metabolism towards aerobic fermentation supporting ATP production and promoting epimastigotes proliferation inside the vector.

## Introduction

Chagas disease, or American trypanosomiasis, is caused by *Trypanosoma cruzi* parasites [1]. In South and Central America, it is a major parasitic disease and worldwide it is the third major parasitic disease after malaria and schistosomiasis. Currently, it is estimated that seven to eight million people are infected worldwide, especially in Latin America where it is endemic [2], and the main cause of non-ischemic heart diseases, costing 1.2 billion dollars annually [3]. Therefore, Chagas disease stands out as a public health problem not only in Latin America but also in other continents, mainly North America and Europe. This spreading is mainly due to immigration of infected individuals and the insect vector expansion over border areas [4]. The life cycle of *T*. *cruzi* involves two hosts and at least four stages: amastigotes and bloodstream trypomastigotes in the mammalian host, and epimastigotes and metacyclic trypomastigotes in the triatomine insect. Each host imposes various conditions and environments to the parasite resulting in the proliferation and differentiation processes along the life cycle that involve modifications in metabolic pathways and gene expression in these organisms [5, 6]. Therefore, the adaptation to environmental changes represents an important coevolution mechanism to thrive inside the insect vectors. In this context, the heme molecule is an example of this intimate relationship between parasite and the insect because it is capable of promoting the proliferation of epimastigote forms [7, 8, 9, 10].

Heme (ferriprotoporphyrin-IX) is an iron-containing porphyrin that participates in many biological reactions, including electron transport, detoxification and oxygen transport [11, 12], which are processes that are essentially mediated by heme proteins such as mitochondrial cytochromes, catalase, and hemoglobin. The heme molecule increases greatly the proliferation of *T*. *cruzi* epimastigotes participating in cell signaling processes that involves a calcium calmodulin kinase II-like [9]. However, unlike most organisms, *T*. *cruzi* lacks complete and canonic routes for heme synthesis and degradation to biliverdin, requiring its incorporation from the environment and different mechanisms for maintaining its homeostasis [13, 14].

Regarding energy metabolism, trypanosomatids present several distinct features such as a single mitochondrion, that present different energetic and antioxidant enzymes with specific arrangement of mitochondrial DNA (kinetoplast) [15, 16], and glycosomes, peroxisome-like organelles that compartmentalize essential metabolic pathways such as glycolysis, gluconeogenesis, purine salvage, pyrimidine biosynthesis, sugar-nucleotide biosynthesis, pentose phosphate pathway, and oxidative stress defense functions [17]. *T cruzi* has shown large variation on its energy metabolism along the life cycle; in the presence of glucose, epimastigotes preferentially catabolized this carbon source whereas in the absence of glucose, amino acids are the preferred energy sources, especially in the stationary growth phase in which amino acids are oxidized to maintain the redox balance and energy [18]. Bloodstream trypomastigotes however seem to rely more on glycolysis rather than oxidative phosphorylation to obtain energy [19], while amastigotes present no detectable glucose transport and therefore are dependent on lipid-based energy metabolism [20].

*T*. *cruzi* possess some distinctive features such as the unique mechanisms for gene expression with the absence of classical promoters for RNA polymerase II, trans-splicing and the organization *in tandem* of genes not necessarily related in function, resulting in polycistronic transcription of genes that are transcribed and processed constitutively. Also, gene expression is mainly regulated post-transcriptionally relying on selective transport to the cytoplasm and mRNA stability [21]. The mechanisms that regulate stage-specific gene expression in this parasite have become better defined after the whole genome sequence became available and the demonstration that at least half of *T*. *cruzi* genes are differentially regulated during its life cycle [22].

Therefore, understanding the transcriptome is an essential initial step for interpreting the functional elements of the genome and for understanding the development of the disease. The high-throughput RNA sequencing (RNASeq) method promotes both mapping and quantification of the entire transcriptomes pool [23]. Thus, the RNA-Seq analysis should be a valuable screening tool strategy for identifying stage-regulated *T*. *cruzi* genes and metabolic pathways affected by heme. Due to the reduced amount of information available on the heme metabolism in protozoa and the essential character of the heme molecule for the *T*. *cruzi* biology, in the present work we identified genes differentially expressed by epimastigotes cultured in media supplemented with heme. Our data suggests that heme modulates epimastigotes energy metabolism by increasing the expression of glycolysis genes in detriment to those of oxidative phosphorylation, shifting the energy metabolism towards aerobic fermentation to induce epimastigote adaptation and proliferation inside the vector.

## Results

### Heme differently regulates transcripts levels of epimastigotes

**Fig 1:**
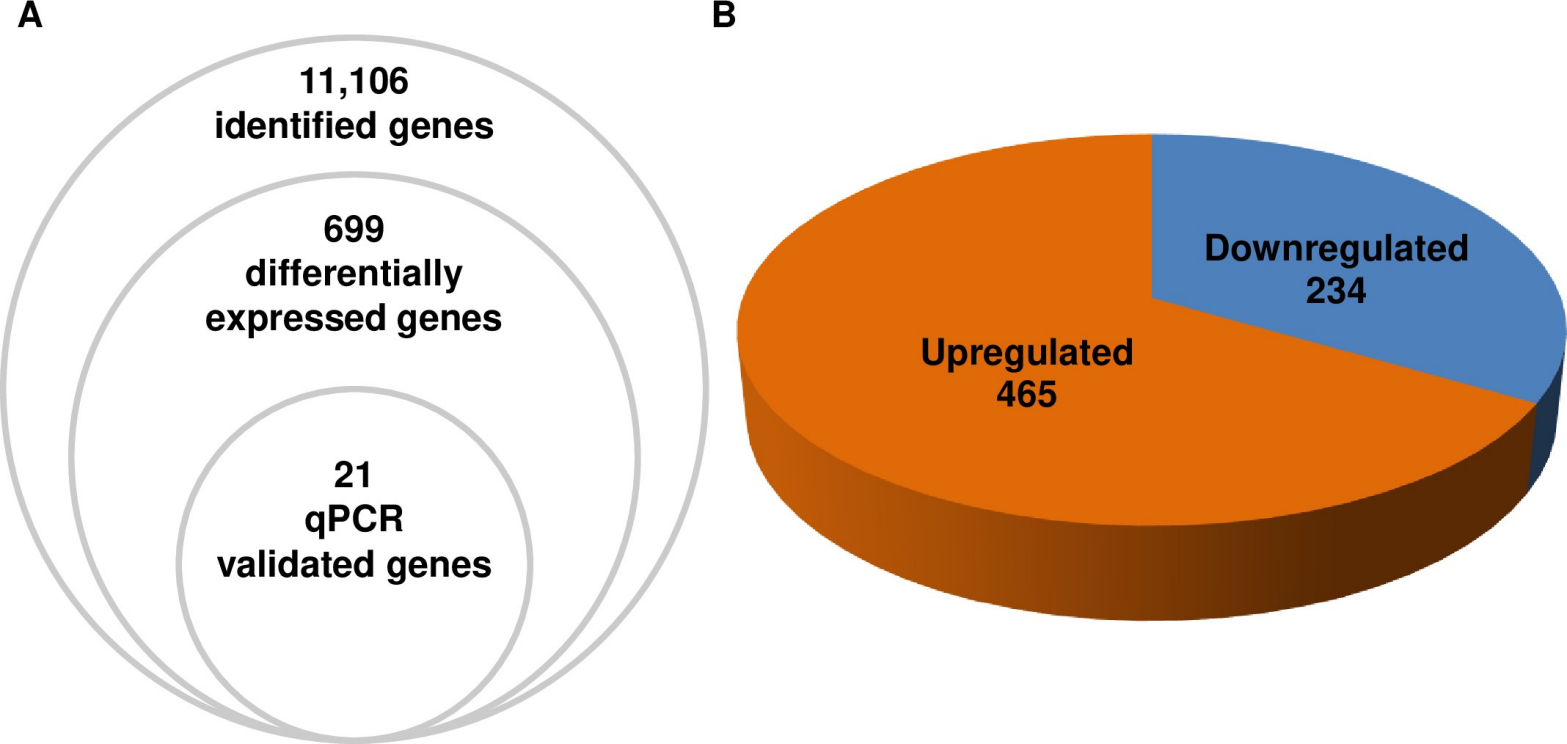
Differentially expressed genes in response to heme treatment. **(A)** RNAseq analysis showing the number of genes identified and differentially expressed. **(B)** Upregulated and downregulated genes modulated by heme.

The impact of the trypanosomatid genome sequencing projects, especially *Trypanosoma brucei*, *Trypanosoma cruzi*, and *Leishmania* species, has been substantial [13]. However, a substantial number of hypothetical trypanosomatid-specific genes remain uncharacterized regarding their possible roles in infectivity and disease. As heme is an important signalling molecule for *Trypanosoma cruzi* [8], we analysed the transcriptome profiling of epimastigotes supplemented with 30 μM heme in order to study the differential expression of genes during its metabolism. PolyA^+^RNA from parasites were purified and RNA-seq was used for this analysis. In order to confirm the haplotype to be used as reference, the reads were mapped separately to both CL Brener haplotypes. The analysis of epimastigotes forms identified 10,596 and 11,106 genes from the annotated genomes of CL Brener Esmeraldo (ESM) and non-Esmeraldo like (NES) haplotype respectively, both filed at TriTrypDB (S1 and S2 Tables). The discrete typing unit of the organism used in this experiment was TcVI; this organism is reported to contain approximately 12,000 genes [13] and is of a hybrid nature, hence the close similarity in the number of mapping results between Esmeraldo and non-Esmeraldo like. Based on these results and to avoid gene duplications, the CL Brener non-Esmeraldo like haplotype was selected as references and used in subsequent analysis. Table 1 shows the samples that were constructed: C1 and C2 (control samples) and H1 and H2 (parasites supplemented with heme). For all samples, the sequencing resulted in more than 25 million clean reads for each sample and more than 83% of reads were counted and mapped in the reference genome. More than 55% of total reads showed to be uniquely and correctly mapped to the reference genome, corroborating the quality of the sequencing for further analysis (Table 1). An adjusted *p-*value ≤ 0.05 was used to select genes that exhibited significant differential expression (S1 Table). Compared to control parasites, heme-treated epimastigotes presented 699 differentially expressed genes (DEGs) of which 465 genes (66,5%) were upregulated and 234 genes (33,5%) were downregulated (S1 Table; Fig 1). A subset of 21 genes of DEGs was selected for posterior qPCR validation.

**Table 1:** Illumina deep sequencing reads map. RNA was extracted from samples by using TRIzol method (Invitrogen) and its degradation and contamination were monitored on 1% agarose gels. The cDNA synthesis was performed with 3 μg of total RNA using NEBNext Ultra RNA Library Prep Kit for Illumina (NEB, USA) following the manufacturer's recommendations. The Deep Seq transcriptome sequencing analysis was carried out using polyA selected RNA at 30M PE reads per sample in an Illumina NextSeq500 sequencer. Reference genomes and gene model annotation files were used from TriTryp database. Raw sequencing reads (raw data) were processed and the read counts were extracted. The raw reads were then filtered to remove low-quality sequences and empty reads to gain clean reads. All subsequent analyses were based on the clean reads with high quality.

Sample	Total trimmed PE reads	Total PE mapped Reads	Total unique and correctly mapped PE reads	Assigned PE reads	Assigned to PC genes
**C1**	31,141.513	24,464.058 (85%)	17,381.971 (55.8%)	14,539.706 (83.7%)	14,444.042 (83.1%)
**C2**	40,580.713	34,586.942 (85.2%)	23,772.450 (58.6%)	19,875.445 (83.6%)	19,750.910 (83.1%)
**H1**	30,751.219	25,821.799 (84%)	17,092.796 (55.6%)	14,085.821 (82.4%)	13,919.867 (81.4%)
**H2**	29,954.970	25,159.179 (84%)	16,601.161 (55.4%)	13,637.847 (82.2%)	13,493.244 (81.3%)

PE, paired-end; PC, protein-coding

### GOs are modulated in heme-treated epimastigotes

**Fig 2:**
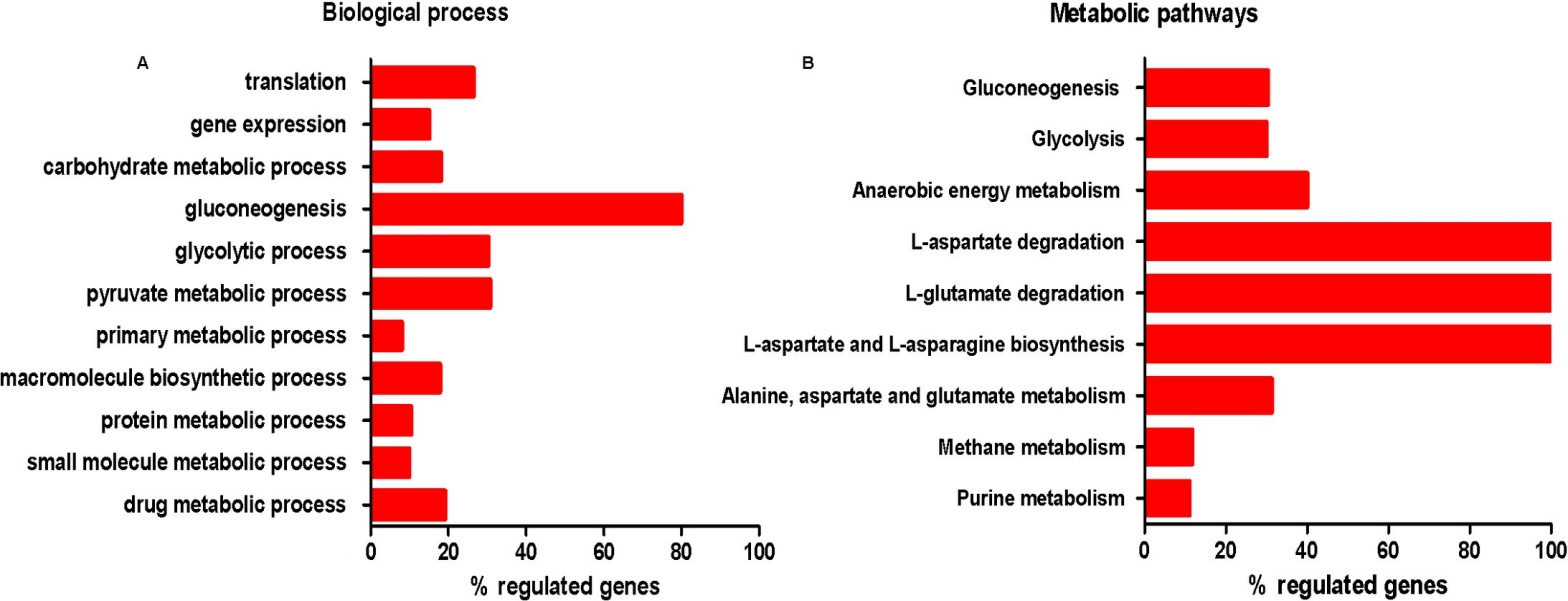
Gene ontology (GO) classification, KEGG/MetaCyc analysis of the differentially expressed genes (DEGs) of heme-treated parasites. The percentage regulated genes based on GO annotations and KEGG/MetaCyc pathways. The **(A)** biological process and **(B)** metabolic pathways. % regulated genes is the percentage of the background or GO/pathway-related genes that were observed as differentially expressed in RNA-Seq data. FDR ≤ 0,05.

After DEGs identification, we performed the GO annotation. The DEGs were enriched as biological processes (Fig 2A) and metabolic pathways (Fig 2B). Both GO terms and pathways were considered statistically significant when they possessed False Discovery Rate (FDR) less than or equal to 0.05. The results showed that gluconeogenesis (Percentage of Background, 80%; Fold Enrichment Value, 14.8; P-value, 4.08E-05), glycolytic (Percentage of Background, 30.4%; Fold Enrichment Value, 5.6; P-value, 0.0), and pyruvate metabolic processes (Percentage of Background, 30.8%; Fold Enrichment Value, 5.7; P-value, 4.57E-05) were the most regulated biological processes stimulated by heme among upregulated genes (Fig 2A, S1 Table). The metabolic pathways most upregulated by heme in epimastigotes were related to L-glutamate and L-aspartate metabolisms (Percentage of Background, 100%; Fold Enrichment Value, 22.0; P-value, 4.21E-06), anaerobic energy metabolism (Percentage of Background, 40%; Fold Enrichment Value, 8,7; P-value, 7.1E-04), gluconeogenesis (Percentage of Background, 30.4%; Fold Enrichment Value, 6.6; P-value, 4.69E-05) and glycolysis (Percentage of Background, 30%; Fold Enrichment Value, 6.5; P-value, 1.9E-04) among the upregulated genes. (Fig 2B; S1 Table).

### Heme modulates the expression of energy metabolism genes

**Fig 3:**
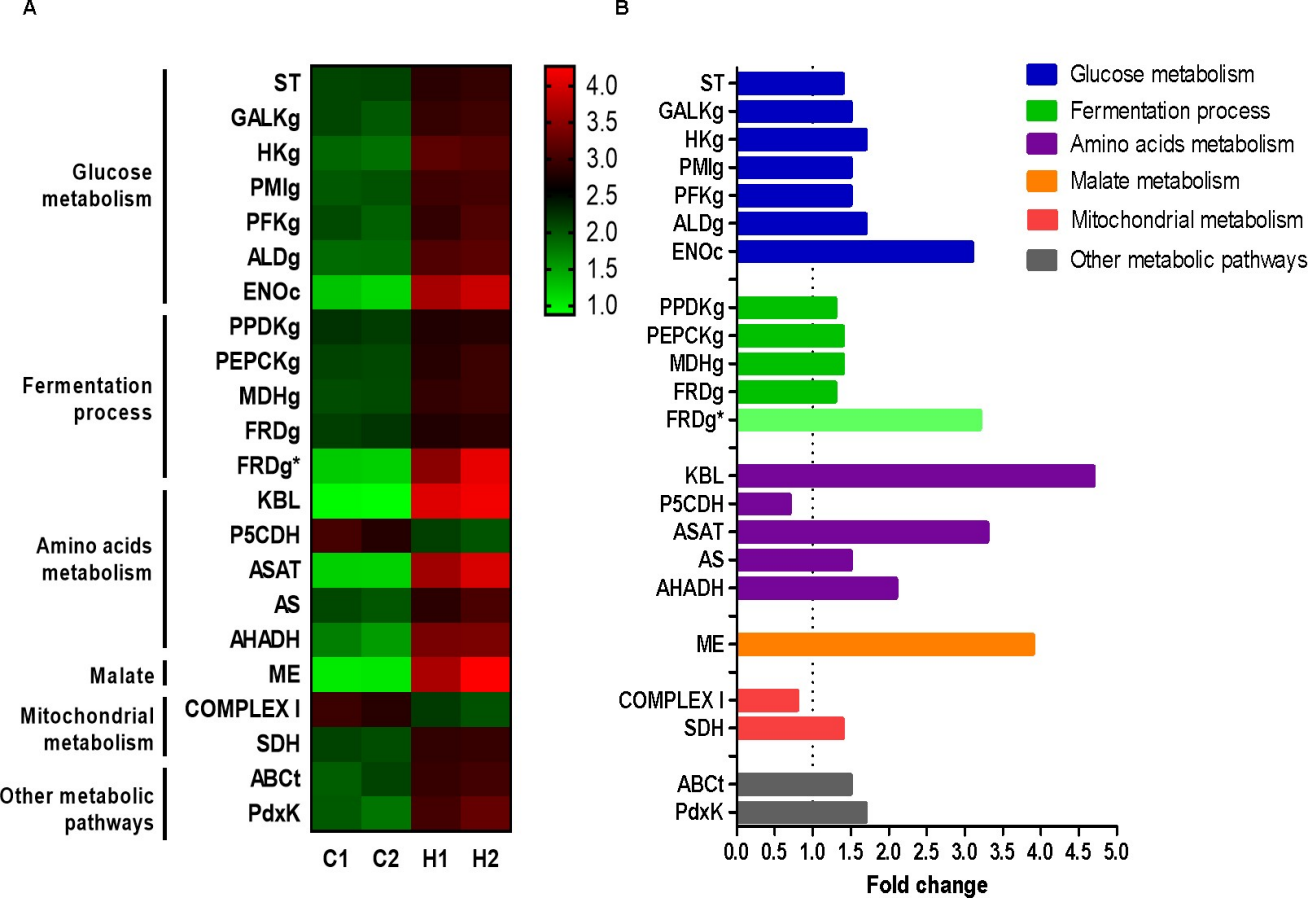
Differently expressed genes related to energy metabolism in epimastigotes. **(A)** RNAseq heatmap of selected genes expressed of control and heme treated samples. Columns represent each technical replicate from two independent experiment. The colors vary from light green (very low expression) to light red (extremely high expression). Data were scaled by the ratio of FPKM of each sample by the total of gene multiplied by 10. **(B)** Transcripts levels of energy metabolism genes presented as fold change compared to control samples. **ST:** sugar transporter; **GALKg**: galactokinase, glycosomal **HKg**: hexokinase, glycosomal; **PMIg:** phosphomannose isomerase, glycosomal; **PFKg**: phosphofructokinase, glycosomal; **ALDg**: fructose bisphosphate aldolase, glycosomal; **ENOc:** enolase, cytosolic; **PPDKg**: pyruvate phosphate dikinase, glycosomal; **PEPCKg**: phosphoenolpyruvate carboxykinase, glycosomal; **MDHg**: malate dehydrogenase, glycosomal; FRDg^*^: NADH-dependent fumarate reductase, glycosomal; **KBL**: 2-amino-3-ketobutyrate CoA ligase; **P5CDH**: δ-1-pyrroline-5-carboxylate dehydrogenase; **ASAT:** aspartate aminotransferase; **AS**: asparagine synthetase A; **AHADH:** aromatic L-2-hydroxyacid dehydrogenase; **ME**: malic enzyme; **COMPLEX I**: NADH-ubiquinone oxidoreductase; **SDH**: succinate dehydrogenase subunit; **ABCt**: ABC transporter; **PdxK**: pyridoxal kinase. These graphs present DEGs in “non-esmeraldo- like” haplotype and some exclusive in “esmeraldo-like” were marked with (*).

Using bioenergetics assays, Nogueira *et al*. (2017) recently showed that heme alters the mitochondrial physiology in epimastigotes. However, the levels of ATP remained the same regardless of the presence of heme [24]. The analysis of NES heme-treated genes demonstrated that this haplotype presented an upregulation in genes involved in the glucose metabolism when compared to mitochondrial metabolism (S1 Table). Hence in order to compare our transcriptomic data with their findings we selected for further analysis 21 genes involved predominantly in energy metabolism. During this process one substantial difference between haplotypes ESM and NES (S1 and S2 Tables) for fumarate reductase genes was unexpectedly observed. Since both haplotypes are expected to be present in the CL Brener strain populations in nature, we showed these discrepancies for better biological analysis. The heatmap from RNA sequencing data of 21 genes are shown in Fig 3A. These observations can be corroborated with Fold Change analysis which also shows an increment in the expression of genes involved in glucose metabolism as 1.44-fold for the sugar transporter, 1.7-fold for hexokinase, and 3.1-fold for enolase (Fig 3B). Furthermore, *T*. *cruzi* treated with heme showed 1.5-fold for phosphofructokinase and 1.7-fold for fructose biphosphate aldolase. Surprisingly, the expression of galactokinase (1.46-fold) and phosphomannose isomerase (1.5-fold) are also increased highlighting the parasite consumption of various monosaccharides as carbon sources other than glucose (Fig 3B).

Additionally, genes related to aerobic fermentation present in the glycosomes such as: pyruvate phosphate dikinase (1.3-fold), phosphoenolpyruvate carboxykinase (1.4-fold), malate dehydrogenase (1.4-fold) and NADH fumarate reductase (1.3-fold for NES and 3.1-fold for ESM) were also upregulated in heme-treated epimastigotes (Fig 3B).

Epimastigotes treated with heme also presented an increased expression of enzymes involved in amino acid metabolism, such as such as aspartate aminotransferase (3.3-fold), asparagine synthetase A (1.5-fold), and alpha-hydroxy acid dehydrogenase (2.1-fold). Heme also modulated the gene expression of 2-amino-3-ketobutyrate CoA ligase (4.7-fold) and δ-1-pyrroline-5-carboxylate dehydrogenase (0.7-fold), mitochondrial enzymes involved in amino acids metabolism (Fig 3B).

Besides NADH-ubiquinone oxidoreductase gene (0.8-fold) and succinate dehydrogenase (1.40-fold) (Fig 3B; S1 Table), most of the other genes involved in mitochondrial energy metabolism did not show any significant alteration in expression in response to heme. These results are in agreement with recent bioenergetic assay findings showing an impairment of mitochondrial oxidative phosphorylation in heme-treated epimastigotes [24]. The malic enzyme was upregulated in RNA-seq assay (3.9-fold) that highlight the relevance of malate metabolism to heme-treated parasite (Fig 3B).

### Glycosomal metabolism and the succinic fermentation in the presence of heme

**Fig 4:**
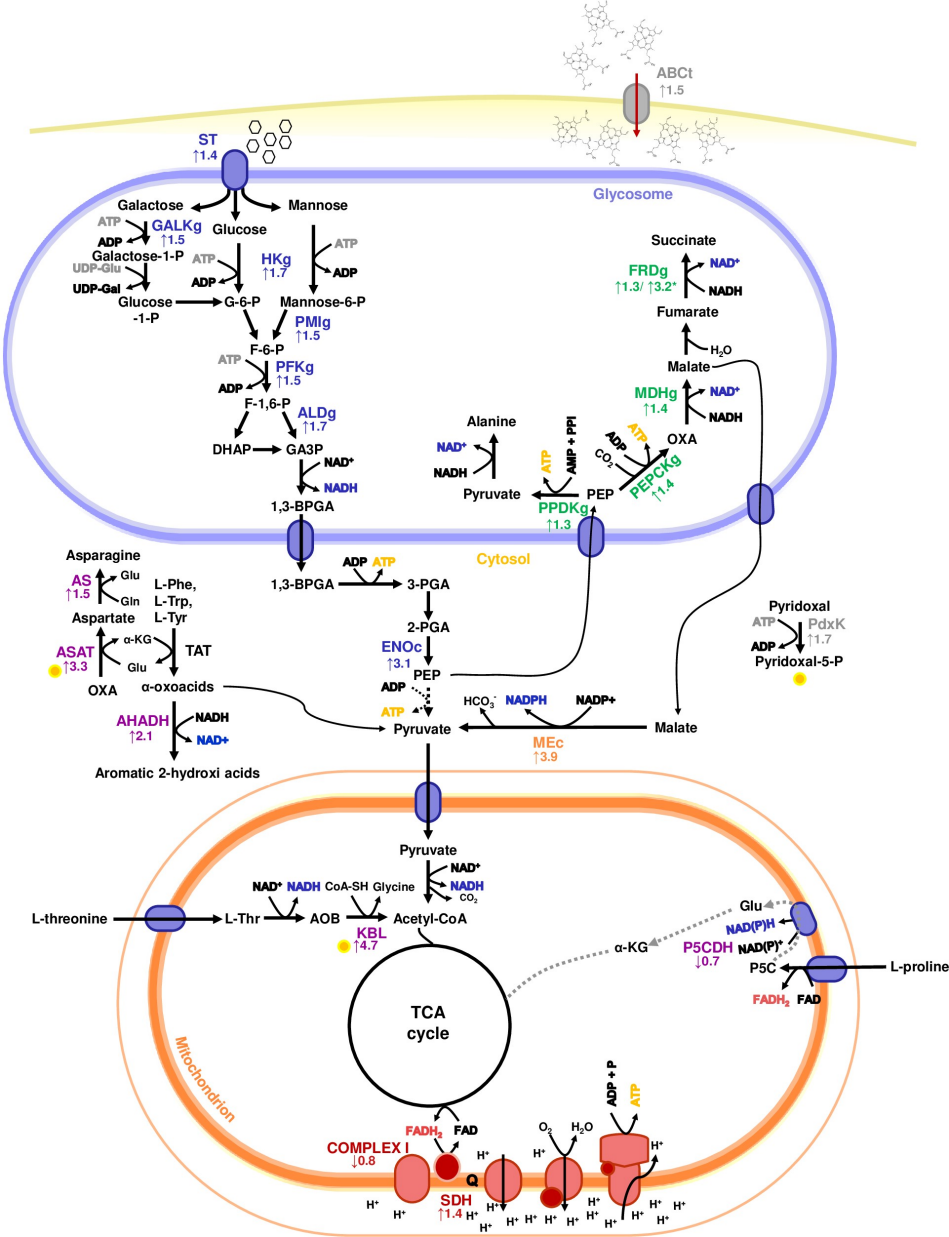
Schematic representation of the metabolic map of gene expression regulated by heme. *Trypanosoma cruzi* epimastigotes metabolic pathway illustrating differentially expressed genes in response to heme treatment involved in energy metabolism measured by RNAseq analysis. Values inbox are expressed as fold change of heme treated parasites in relation to control sample. Enzymes reactions were presented as known (black solid arrow), downregulated suggested-routes (black dashed arrow) and downregulated (gray solid arrow) routes by RNAseq data. Yellow circles indicate pyridoxal 5-phosphate binding enzymes. The enzymes of each proposed pathway are presented as specific colors. **Glucose metabolism (blue): ST:** sugar transporter; **GALKg**: galactokinase, glycosomal; **HKg**: hexokinase, glycosomal; **PMIg:** phosphomannose isomerase, glycosomal; **PFKg**: phosphofructokinase, glycosomal; **ALDg**: fructose bisphosphate aldolase, glycosomal; **ENOc:** enolase, cytosolic. **Fermentation process (green): PPDKg**: pyruvate phosphate dikinase, glycosomal; **PEPCKg**: phosphoenolpyruvate carboxykinase, glycosomal; **MDHg**: malate dehydrogenase, glycosomal; FRDg*: NADH-dependent fumarate reductase, glycosomal. **Amino acids metabolism (purple): KBL**: 2-amino-3-ketobutyrate CoA ligase; **P5CDH**: δ-1-pyrroline-5-carboxylate dehydrogenase; **ASAT:** aspartate aminotransferase; **AS**: asparagine synthetase A. **AHADH:** aromatic L-2-hydroxyacid dehydrogenase; **Malate metabolism (orange): ME**: malic enzyme. **Mitochondrial metabolism (pink): COMPLEX I**: NADH-ubiquinone oxidoreductase; **SDH**: succinate dehydrogenase subunit. **Other metabolic pathways (gray): PdxK**: pyridoxal kinase; **ABCt**: ABC transporter. Metabolytes: G-6-P: glucose-6-phosphate; F-6-P: fructose-6-phosphate; F-1,6-P: fructose-1,6-biphosphate; DHAP: dihydroxyacetone phosphate; **GA3P**: glyceraldehyde-3-phosphate; 1,3-BPGA: 1,3-bisphosphoglycerate; 3-PGA: 3-phosphoglycerate; 2-PGA: 2-phosphoglycerate; PEP: phospho*enol*pyruvate; OXA: oxaloacetate; Glu: glutamate; Gln: glutamine; Phe: phenylalanine; Trp: tryptophan; Tyr: tyrosine; P5C: δ-1-pyrroline-5-carboxylate; **AOB**: amino-oxobutyrate.

It is well documented in the literature that *Trypanosoma cruzi* major products of aerobic fermentation processes are succinate, alanine and acetate, [25]. Fig 4 summarizes the schematic representation of the proposal succinic fermentation pathway since three of four main glycosomal enzymes are upregulated by heme phosphoenolpyruvate carboxykinase (1.4-fold), malate dehydrogenase (1.4-fold) and NADH-dependent fumarate reductase (1.3-fold for NES and 3.2-fold for ESM). This concept is reinforced by the upregulation of the malic enzyme (3.9-fold), producing pyruvate or fumarate, a succinic fermentation intermediary that returns to glycosome. Additionally, pyruvate phosphate dikinase gene increased 1.3-fold, a glycosomal enzyme that converts phosphoenolpyruvate (PEP), inorganic pyrophosphate (PPi) and AMP into pyruvate, inorganic phosphate (Pi) and ATP additionally supporting fermentation [26] was also upregulated by heme. Thus, RNA-seq results suggest that heme controls epimastigote transcript abundance inducing metabolic changes in the parasite in order to improve glycolysis and fermentation that ultimately will promote epimastigote proliferation and establishment inside the vector.

**Fig 5:**
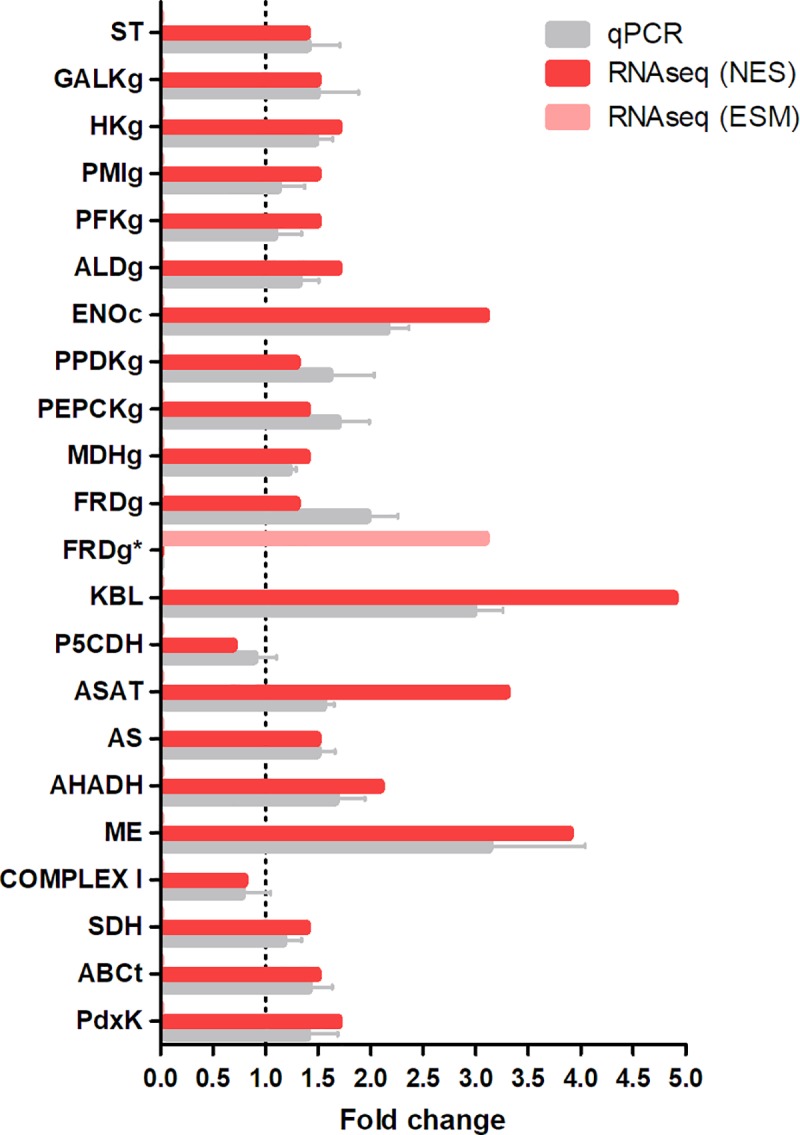
RNAseq and qPCR data of selected genes modulated by heme. For qPCR validation, total RNA was extracted using TRIzol method (Invitrogen) and then was reverse-transcripted to single strand cDNA using High Capacity cDNA Reverse Transcription Kit (Applied Biosystems, USA) according to the manufacturer's instructions. All quantitative measurements were carried out in triplicate and normalized to the internal *TCZ* control. Results were expressed as mean value ± standard error (SE). The mRNA fold change was calculated as described in the Material and Methods section. qPCR and RNAseq data were plotted for each gene for analysis. **ST:** sugar transporter; **GALK**: galactokinase; **HK**: hexokinase; **PMI:** phosphomannose isomerase; **PFK**: phosphofructokinase; **ALD**: fructose bisphosphate aldolase, glycosomal; **ENO:** enolase; **PPDKg**: pyruvate phosphate dikinase, glycosomal; **PEPCKg**: phosphoenolpyruvate carboxykinase, glycosomal; **MDHg**: malate dehydrogenase, glycosomal; FRD*: NADH-dependent fumarate reductase; **KBL**: 2-amino-3-ketobutyrate CoA ligase; **P5CDH**: δ-1-pyrroline-5-carboxylate dehydrogenase; **ASAT:** aspartate aminotransferase; **AS**: asparagine synthetase A; **AHADH:** aromatic L-2-hydroxyacid dehydrogenase; **ME**: malic enzyme, cytosolic; **COMPLEX I**: NADH-ubiquinone oxidoreductase; **SDH**: succinate dehydrogenase subunit; **ABCt**: ABC transporter; **PdxK**: pyridoxal kinase. This graphic presents DEGs in “non-esmeraldo-like” haplotype and some only in “esmeraldo-like” that were marked with (*).

In order to validate the RNAseq results in heme-treated epimastigotes of *T*. *cruzi* we selected 21 genes and carried out qRT-PCRs. Triplicates of cDNA samples were used for each experimental group and all the selected genes were assayed by at least five independent replicates as described in the Material and Methods section. As expected, the qPCR results corroborated to the data from the RNAseq data analysis (Fig 5).

## Discussion

Protozoan parasites have evolved diverse metabolic strategies for surviving and proliferating within different extracellular and intracellular niches in their hosts. It is widely known that environmental cues are linked to the differentiation process that allows the parasite to complete its life cycle. Even if challenging, successful transmission depends on parasite ability to trigger adaptive responses and cope with stressors whilst regulating proliferation and transition to different life stages [27]. During the journey of epimastigote forms in the invertebrate host, *T*. *cruzi* must survive within the digestive constraints of the triatomine gut [28]. The triatomine insect ingests a huge amount of blood, releasing in the midgut lumen sugars, lipids, peptides, amino acids and almost 10 mM heme products of the blood digestion [29]. In the midgut medium, the parasite shows an important adaptation to the redox status of the environment and its nutritional availability given that the nutrients are now disputed with the host and the insect microbiota [27, 30, 31].

In the present work we performed a transcriptomic analysis using RNAseq technology and validate the results of 21 genes by qPCR to study the metabolic changes that heme, an abundant molecule present in the insect vector, induced in epimastigotes to sustain the parasite proliferation. *T*. *cruzi* must develop a broad set of molecular tools that allow it to multiply in the insect gut, to invade and multiply inside a large number of distinct mammalian cell types and to circumvent host immune defense systems. To meet such phenotypic plasticity, *T*. *cruzi* relies on unique mechanisms that can control the expression of its repertoire of thousands of genes. Because its genome is constitutively transcribed into long polycistronic primary transcripts, mRNAs for protein-coding genes must be processed through trans-splicing and polyadenylation reactions [21]. The mRNAs must also interact with different protein factors in complex post-transcriptional regulatory machinery that determines the levels of their protein products according to the cellular demands of the parasite in each stage of the life cycle [22].

Although much progress has been made in identifying the molecular mechanisms that determine parasite host cells and tissues invasion and evasion of the host immune response, much less is known about the exchange of nutrients and the metabolic interplay that occurs between *T*. *cruzi* and the insect vector environment. In all life cycle stages, the mitochondrion of *T*. *cruzi* is aerobically functioning, however pyruvate is not oxidized to carbon dioxide, and instead, fermentation-like end products, such as acetate, alanine and succinate are produced. In fact, many aspects of trypanosomatid metabolism were initially deduced by analysis of secreted end-products using classic biochemical methods in *Trypanosoma brucei*. Levels of some intracellular purines and pyrimidine nucleotides [32] or thiamine and its precursors were studied [33]. Early studies also investigated the utilization of different carbon sources and nutrients by *Leishmania mexicana* promastigotes and demonstrated that in the exponential phase of proliferation these parasites increase the uptake of glucose and amino acids. The rate of use of these metabolites decreased in the promastigotes stationary phase and axenic amastigotes, consistent with the latter stages entering a reduced metabolic state. Stationary phase promastigotes and axenic amastigotes also oxidise free fatty acids to drive mitochondrial oxidative phosphorylation and ATP production [34].

Trypanosomatids have a high rate of glucose consumption and this is associated with aerobic fermentation of glucose [35]. The mitochondrial energy metabolism of trypanosomatids can be considered an intermediate between classical aerobically functioning mitochondria and true anaerobically functioning mitochondria, similar to most parasitic helminths and several marine organisms [36].

Our approach used the high-throuput RNAseq technology to identify the main expressed genes induced by heme in *T*. *cruzi* epimastigotes. Remarkably, the main biological processes stimulated by heme were fermentation related enzymes (malic enzyme, fumarate reductase and glycosomal malate dehydrogenase isoforms) and genes involved in amino acid metabolism. This increase in the amino acid metabolism was expected since it is well stablished that epimastigotes can use either amino acids or glucose as a carbon source for growth, although glucose is preferred during the exponential phase and amino acids preferred during the stationary phase [35, 37, 38, 39]. Interestingly, heme-induced epimastigote proliferation is accompanied by an increased number of cells in S and G2/M phases, keeping the parasites in the exponential growth phase for longer period of time. This shows a modulation of *T*. *cruzi* cell cycle by heme [30]. These finds corroborated metabolomic data from Barisón and collaborators (2017) that demonstrated metabolic plasticity of epimastigotes, which are able to switch the use of nutrients depending on its abundance. Notably both stationary and exponential phases epimastigotes maintained intracellular levels of glucose using amino acids for *de novo* synthesis of glucose or by arrested glucose consumption [18]), confirming the central role of glucose as the “fuel of life” on epimastigote metabolism. Many organisms that face environmental hypoxia such as marine invertebrates, *Fasciola*, *Ascaris lumbricoides*, *and Blastocystis sp*, experience a shift in the energy metabolism from oxidative phosphorylation to a fermentative route of carbohydrates degradation named malate dismutation. In this pathway phosphoenolpyruvate (PEP) from glycolysis is converted to oxaloacetate via an ATP-linked PEP carboxykinase (ATP-PEPCK), which is subsequently reduced by cytosolic malate dehydrogenase (MDH), re-oxidizing the glycolytic NADH. Malate is then, imported into the mitochondrion, where it is degraded via malate dismutation. A portion of the malate is oxidized to acetate and another portion is reduced to succinate, which is then further metabolized to propionate [40]. Interestingly, this metabolic plasticity was also reported in *T*. *brucei* [41].

In this work transcriptomic analysis showed that heme increased the expression of glycosomal enzymes involved in glucose/hexoses metabolism. It is described that the main end products of aerobic fermentation process in glycosomes are succinate and alanine and both can be produced via phosphoenolpyruvate (PEP) produced in the cytosol [35]. Our data have shown that heme induced the upregulation of the hexokinase, PFK and enolase, inducing the glycolytic pathway and increasing the cytosolic concentrations of PEP. It is generally accepted that PEP has at least three destinations in the parasite, two inside the glycosome and one in the cytosol. Within the glycosome phosphoenolpyruvate may have two fates: **(i)** to be converted into pyruvate by a pyrophosphate dependent pyruvate phosphate dikinase (PPi-dependent PPDK) or, **(ii)** PEP can be carboxylated to oxaloacetate by the ADP-dependent PEPCK, yielding ATP in both cases and leading afterwards to NADH oxidation. [42]. A third destination for PEP is the cytosol, following the glycolytic pathway where it can be converted into pyruvate by the cytosolic pyruvate kinase, also yielding ATP.

Our data demonstrates that heme upregulated the expression of both PPDK and the PEPCK. Since heme-treated epimastigotes also presented an upregulation of the glycosomal malate dehydrogenase and fumarate reductase and had no effect over the expression of alanine dehydrogenase, we hypothesize that the PEP is being preferably carboxylated into oxaloacetate by glycosomal PEPCK yielding great amounts of succinate and NAD^+^, a pathway known as succinic fermentation [43]. Interestingly, cytosolic glyceraldehyde-3-phosphate dehydrogenase (GAPDHc) and glycosomal enzyme (GAPDHg) in the presence of heme were not affected, suggesting the ratio of NADH/NAD^+^ maintenance in the glycosome by other glycosomal dehydrogenases.

Remarkably one of the most upregulated enzymes was malic enzyme. This enzyme produces pyruvate and NADPH and is considered crucial for gluconeogenesis, glycolysis, and fatty acid synthesis [44]. It was suggested that malic enzyme is important for NADPH production especially in *T*. *cruzi* forms that have low glucose availability, reducing PPP activity [45]. Here, our results suggest that epimastigotes consume glucose in the fermentation process which could reduce the consumption of glucose in PPP. Therefore, the increase in malic enzyme activity could maintain NADPH levels in epimastigotes forms. Therefore, the increase in malic enzyme activity could maintain NADPH levels in epimastigotes forms.

Compared to other evolutive forms, *T*. *cruzi* epimastigotes generally have an increased gene expression related to the TCA cycle, respiratory chain and oxidative phosphorylation, suggesting a rise in the overall respiratory activity [46]. However, previous biochemical analysis already showed that heme compromises mitochondrial physiology decreasing the activity of the respiratory system through the hyperpolarization of the mitochondrial membrane and consequent overproduction of superoxide anion. Surprisingly, despite these effects, the parasite is able to maintain constant intracellular ATP levels [24].

Even though fermentation is classically considered less efficient compared to cellular respiration, glucose preference provides a prompt carbon source for metabolic biosynthesis pathways, mainly amino acids, lipids and nucleic acids. Furthermore, part of glucose can be oxidized into CO_2_ by the pentoses phosphate pathway (PPP) in order to provide NADPH and ribose-5-phosphate required for anabolic processes and replication [25]. This reinforces our hypothesis of the influence of heme in the shift of *T cruzi* epimastigote energy metabolism and that the contribution in ATP production may be based on glycosomal fermentation process, providing energy support for the proliferation and establishment of the parasite inside the vector.

## Materials and methods

### Cultivation of microorganism

Epimastigote forms of *Trypanosoma cruzi*, clone CL Brener were grown in 50 mL medium BHI with 10% FCS in the presence of 30 μM heme to 28°C for 7 days in culture bottle cells with 25 cm^2^ surface area (TECHNO PLASTIC PRODUCTS AG, Switzerland).

### Heme

20 mM heme stock solution was freshly prepared by dissolving in 0.1 N NaOH, and after it was buffered in PBS (100 mM sodium phosphate buffer and 150 mM NaCl at pH 7.4). The stock solution was diluted immediately before use to 5 mM in the same PBS buffer or in culture medium.

### RNA isolation, library construction and Illumina sequencing processing

RNA samples of epimastigotes incubated with or without heme were extracted from samples by using TRIzol method (Invitrogen, USA). Total RNA was measured using Qubit RNA assay kit (Life technologies, USA). The mRNA enrichment was performed in a NEBNext Poly(A) mRNA Magnetic Isolation Module (NEB, UK). cDNA libraries were synthesized by Illumina TruSeq RNA Sample Preparation Kit. The final library had an insert size about 150 bp. The Deep Seq transcriptome sequencing analysis was carried out using polyA selected RNA at 30M Paired Ended reads per sample in an Illumina NextSeq500 sequencer.

### Bioinformatics analysis

Raw PE reads were trimmed against adaptor sequences by scythe (v0.981), and quality-trimmed by sickle (v1.33) using default settings. Trimmed reads were directionally separately aligned to the haploid genome of *T*. *cruzi* strain CL Brener Esmeraldo and Nonesmeraldo-like available at triTrypDB using bowtie2 (version 2.3.5) with -very-sensitive-local option. Uniquely and correctly mapped reads were extracted for the downstream analysis. Tool feature Counts [47] was used to count the number of reads aligned to each gene. Gene expression level was calculated and normalised by Fragments Per Kilobase Per Million (FPKM). Genes with FPKM < 10 on both conditions were removed from the posterior analysis. The differential expression analysis was performed using the Generalized Fold Change or GFOLD [48] to statistically determine when the fold-change between two conditions is reliable. Absolute fold-change greater or equal than 1.2 and GFOLD ≠ 0 were set as the thresholds for significantly differentially expressed genes (DEGs). Subsequently, the enrichment analysis of Gene Ontology (GO), KEGG and MetaCyC pathways was performed based on these DEGs by using the pipeline of TriTrypDB v44 (https://tritrypdb.org/tritrypdb/) [49]. GO terms and pathways enrichment was considered statistically significant when the *False Discovery Rate* or FDR was less or equal than 0.05.

### qPCR

A subset of differentially expressed genes will be selected to be validated by qPCR. cDNA synthesis was performed using High Capacity cDNA Reverse Transcription Kit (Applied Biosystems, USA) according to the manufacturer's instructions. The qPCR mix was done using a Sybr Green PCR Master Mix (QIAGEN, Germany). Triplicates of cDNA samples were used for each experimental group and all the selected genes were assayed at least five independent replicates. All quantitative measurements were normalized to the internal *TCZ* control for every reaction [50]. Results were expressed as mean value ± standard error (SE). The mRNA fold change was calculated by the following equations: ^Δ^*C*_*T*_ = ^Δ^*C*_*T*(target)_−^Δ^*C*_*T*(TCZ)_; ^ΔΔ^*C*_*T*_ = ^Δ^*C*_*T*(Heme treated)_−^Δ^*C*_*T*(control)_; mRNA fold change = 2^−ΔΔ*C*^_*T*_ [51].

## Supporting information

S1 Table RNA-Seq raw expression of NES; Differential gene expression analysis of NES; Gene Ontology terms enrichment analysis of NES and Pathways enrichment analysis of NES. (XLS)

S2 Table RNA-Seq raw expression of EMS; Differential gene expression analysis of EMS; Gene Ontology terms enrichment analysis of EMS and Pathways enrichment analysis of EMS. (XLS)

## References

[1] Chagas C. New human tripanomíase. Studies on the morphology and the life cycle of *Schizotrypanum cruzi*, n. gen., etiologic agent of new morbid entity of man. *Mem*. *Inst*. *Oswaldo Cruz*. 1909 1: 159–218.

[2] WHO. World Health Organization. Chagas disease (American trypanosomiasis). Fact sheet 2018.

[3] Lee BY, Bacon KM, Connor DL, Willig AM, Bailey RR. The potential economic value of a Trypanosoma cruzi (Chagas disease) vaccine in Latin America, PLoS Negl. Trop. Dis. 2010; 4: e916. 10.1371/journal.pntd.0000916 PMC3001903 21179503

[4] Leslie M. Infectious diseases. Drug developers finally take aim at a neglected disease, *Science*. 2011; 333: 933–935. 10.1126/science.333.6045.933 21852468

[5] Rassi A, Rassi A. Marin-Neto JA. Chagas disease. *The Lancet*. 2010; 375: 1388–402. 10.1016/S0140-6736(10)60061-X 20399979

[6] Bartholomeu DC, de Paiva RMC, Mendes TAO, Da Rocha WD, Teixeira SMR. Unveiling the Intracellular Survival Gene Kit of Trypanosomatid Parasites. *PLoS Pathog*. 2014; 10(12): e1004399 10.1371/journal.ppat.1004399 25474314 PMC4256449

[7] Lara FA, Sant'Anna C, Lemos D, Laranja GAT, Coelho MGP, Reis Salles I, et al Heme requirement and intracellular trafficking in *Trypanosoma cruzi* epimastigotes. *Biochem Biophys Res Commun*. 2007; 355 (1): 16–22. 10.1016/j.bbrc.2006.12.238 17292866

[8] Souza CF, Carneiro AB, Silveira AB, Laranja GAT, Silva-Neto MAC, Gonçalves da Costa SC, Paes MC Heme-induced Trypanosoma cruzi proliferation is mediated by CaMkinase II. Bioch Bioph Res Comm. 2009; 390 (3): 541–546. 10.1016/j.bbrc.2009.09.135 19818332

[9] Nogueira NP, Souza CF, Saraiva FM, Sultano PE, Dalmau SR, Bruno RE, Gonçalves RL, et al MC. Heme-induced ROS in Trypanosoma cruzi activates CaMKII-like that triggers epimastigote proliferation. One helpful effect of ROS. PLoS One. 2011; 6 (10): e25935 10.1371/journal.pone.0025935 22022475 PMC3191175

[10] Paes MC, Cosentino-Gomes D, de Souza CF, Nogueira NP, Meyer-Fernandes JR. The Role of Heme and Reactive Oxygen Species in Proliferation and Survival of *Trypanosoma cruzi*. *J Parasitol Res*. 2011: 174614 10.1155/2011/174614 22007287 PMC3191734

[11] Reddi AR and Hamza I. Heme Mobilization in Animals: A Metallolipid's Journey. *Acc Chem Res*. 2016; 49(6): 1104–10. 10.1021/acs.accounts.5b00553 27254265 PMC5629413

[12] Ponka P Cell biology of heme. *Am*. *J*. *Med*. Sci. 1999; 318: 241–256. 10.1097/00000441-199910000-00004 10522552

[13] El-Sayed NM, Myler PJ, Bartholomew DC, Nilsson D, Aggarwal G, Tran AN et al The genome sequence of *Trypanosoma cruzi*, etiologic agent of Chagas disease. *Science*. 2005; 309 (5733): 409–415. 10.1126/science.1112631 16020725

[14] Cupello MP, Souza CF, Menna-Barreto RF, Nogueira NPA, Laranja GAT, Sabino KCC, Coelho MGP, et al Trypanosomatid essential metabolic pathway: New approaches about heme fate in Trypanosoma cruzi. *Bioch Bioph Res Comm*. 2014; 449: 216–221. 10.1016/j.bbrc.2014.05.004 24824181

[15] Tomás AM, Castro H. Redox metabolism in mitochondria of trypanosomatids. Antioxid Redox Signal. 2013; 19(7): 696–707. 10.1089/ars.2012.4948 23025438 PMC3739956

[16] Cavalcanti DP, de Souza W. The Kinetoplast of Trypanosomatids: From Early Studies of Electron Microscopy to Recent Advances in Atomic Force Microscopy. Scanning. 2018; 2018: 9603051 10.1155/2018/9603051 30018700 PMC6029474

[17] Szöör B, Haanstra JR, Gualdrón-López M, Michels PA Evolution, dynamics and specialized functions of glycosomes in metabolism and development of trypanosomatids. *Curr Opin Microbiol*. 2014; 22: 79–87. 10.1016/j.mib.2014.09.006 25460800

[18] Barisón MJ, Rapado LN, Merino EF, Pral EM, Mantilla BS, Marchese L, Nowicki C, Silber AM, Cassera MB. Metabolomics profiling reveals a finely tuned, starvation-induced metabolic switch in *Trypanosoma cruzi* epimastigotes. *J Biol Chem*. 2017; 292(21): 8964–8977. 10.1074/jbc.M117.778522 28356355 PMC5448128

[19] Gonçalves RL, Barreto RF, Polycarpo CR, Gadelha FR, Castro SL, Oliveira MF. A comparative assessment of mitochondrial function in epimastigotes and bloodstream trypomastigotes of *Trypanosoma cruzi*. *J Bioenerg Biomembr*. 2011; 43: 651–661. 10.1007/s10863-011-9398-8 22081211

[20] Atwood JA 3rd, Weatherly DB, Minning TA, Bundy B, Cavola C, Opperdoes FR, Orlando R, et al The *Trypanosoma cruzi* proteome. *Science*. 2005; 309: 473–476. 10.1126/science.1110289 16020736

[21] Martínez-Calvillo S, Vizuet-De-Rueda JC, Florencio-Martínez LE, Manning-Cela RG, Figueroa-Angulo EE. Gene expression in trypanosomatid parasites. *J Biomed Biotechnol*. 2010; 2010: 525241 10.1155/2010/525241 20169133 PMC2821653

[22] Pastro L, Smircich P, Di Paolo A, Becco L, Duhagon MA, Sotelo-Silveira J. Nuclear Compartmentalization Contributes to Stage-Specific Gene Expression Control in *Trypanosoma cruzi*. *Front Cell Dev Biol*. 2017; 5: 8 10.3389/fcell.2017.00008 28243589 PMC5303743

[23] Wang Z, Gerstein M, Snyder M. RNA-Seq: a revolutionary tool for transcriptomics. *Nat Rev Genet*. 2009; 10(1): 57–63. 10.1038/nrg2484 19015660 PMC2949280

[24] Nogueira NP, Saraiva FMS, Oliveira MP, Mendonça APM, Inacio JDF, Almeida Amaral EE, et al Heme modulates Trypanosoma cruzi bioenergetics inducing mitochondrial ROS production. *Free Rad Biol Med*. 2017; 108: 183–191. 10.1016/j.freeradbiomed.2017.03.027 28363600

[25] Maugeri DA, Cannata JJ, Cazzulo JJ Glucose metabolism in *Trypanosoma cruzi*. *Essays Biochem*. 2011; 51: 15–30. 10.1042/bse0510015 22023439

[26] González-Marcano E, Acosta H, Mijares A, Concepción JL. Kinetic and molecular characterization of the pyruvate phosphate dikinase from *Trypanosoma cruzi*. *Exp Parasitol*.2016; 165: 81–87. 10.1016/j.exppara.2016.03.023 27003459

[27] Jimenez V Dealing with environmental challenges: mechanisms of adaptation in Trypanosoma cruzi. *Res Microbiol* 2014; 165: 155–165. 10.1016/j.resmic.2014.01.006 24508488 PMC3997592

[28] Kollien AH, Schaub GA Development of *Trypanosoma cruzi* after starvation and feeding of the vector—a review. *Tokai J Exp Clin Med*. 1998; 23(6): 335–40. 10622631

[29] Graça-Souza AV, Maya-Monteiro C, Paiva-Silva GO, Braz GR, Paes MC, Sorgine MH, et al Adaptations against heme toxicity in blood-feeding arthropods. *Insect Biochem Mol Biol*. 2006; 36(4): 322–35. 10.1016/j.ibmb.2006.01.009 16551546

[30] Nogueira NP, Saraiva FM, Sultano PE, Cunha PR, Laranja GA, Justo GA, et al Proliferation and Differentiation of Trypanosoma cruzi inside Its Vector Have a New Trigger: Redox Status. *PLoS One*. 2015; 10: e0116712 10.1371/journal.pone.0116712 25671543 PMC4324650

[31] Garcia ES, Genta FA, de Azambuja P, Schaub GA Interactions between intestinal compounds of triatomines and *Trypanosoma cruzi*. *Trends Parasitol*. 2010; 26(10): 499–505. 10.1016/j.pt.2010.07.003 20801082

[32] Fish WR, Looker DL, Marr JJ, Berens RL Purine metabolism in the bloodstream forms of *Trypanosoma gambiense* and *Trypanosoma rhodesiense*. *Biochim Biophys Acta*. 1982; 719: 223–31. 10.1016/0304-4165(82)90092-7 6817814

[33] Stoffel SA, Rodenko B, Schweingruber AM, Maser P, de Koning HP, et al Biosynthesis and uptake of thiamine (vitamin B1) in bloodstream form Trypanosoma brucei brucei and interference of the vitamin with melarsen oxide activity. *Int J Parasitol*. 2006; 36: 229–36. 10.1016/j.ijpara.2005.10.003 16375907

[34] Blum JJ Effects of culture age and hexoses on fatty acid oxidation by Leishmania major. *J Protozool*. 1990; 37: 505–510. 10.1111/j.1550-7408.1990.tb01256.x 2128337

[35] Cazzulo JJ. The aerobic fermentation of glucose by Trypanosomatids. FASEB J. 1992; 6: 3153–3161. 10.1096/fasebj.6.13.1397837 1397837

[36] Tielens AGM, van Hellemond JJ Surprising variety in energy metabolism within Trypanosomatidae. *Trends Parasitol* 2009; 25: 482–490. 10.1016/j.pt.2009.07.007 19748317

[37] Marchese L, Nascimento JF, Damasceno FS, Bringaud F, Michels PAM, Silber AM The Uptake and Metabolism of Amino Acids, and Their Unique Role in the Biology of Pathogenic Trypanosomatids. Pathogens 2018; 7(2): E36 10.3390/pathogens7020036 29614775 PMC6027508

[38] Egel JC, Franke de Cazzulo BM, Stoppani AO, Cannata JJ, and Cazzulo JJ Aerobic glucose fermentation by *Trypanosoma cruzi* axenic culture amastigote-like forms during growth and differentiation to epimastigotes. *Mol*. *Biochem*. *Parasitol*. 1987; 26: 1–10. 10.1016/0166-6851(87)90123-x 3323902

[39] Cazzulo JJ, Franke de Cazzulo BM, Engel JC, and Cannata JJ End products and enzyme levels of aerobic glucose fermentation in trypanosomatids. *Mol*. *Biochem*. *Parasitol*. 1985; 16: 329–343. 10.1016/0166-6851(85)90074-x 3903497

[40] Müller M, Mentel M, van Hellemond JJ, Henze K, Woehle C, Gould SB, Yu RY, van der Giezen M, Tielens AG, Martin WF. Biochemistry and evolution of anaerobic energy metabolism in eukaryotes. *Microbiol Mol Biol Rev* 2012; 76(2): 444–495. 10.1128/MMBR.05024-11 22688819 PMC3372258

[41] Besteiro S, Biran M, Biteau N, Coustou V, Baltz T, Canioni P et al Succinate secreted by *Trypanosoma brucei* is produced by a novel and unique glycosomal enzyme, NADH-dependent fumarate reductase. *J*. *Biol*. *Chem*. 2002; 277: 38001–38012. 10.1074/jbc.M201759200 12138089

[42] Cymeryng C, Cazzulo JJ, Cannata JJ Phosphoenolpyruvate carboxykinase from *Trypanosoma cruzi*. Purification and physicochemical and kinetic properties. *Mol Biochem Parasitol* 1995; 73(1–2): 91–101. 10.1016/0166-6851(95)00099-m 8577351

[43] Michaels PA, Bringaud F, Herman M, Hannaert V. Metabolic functions of glycosomes in trypanosomatids. *Biochim Biophys Acta*. 2006; 1763: 1463–1477. 10.1016/j.bbamcr.2006.08.019 17023066

[44] Spaans SK, Weusthuis RA, van der Oost J, Kengen SW. NADPH-generating systems in bacteria and archaea. *Front Microbiol*. 2015; 6: 742 10.3389/fmicb.2015.00742 26284036 PMC4518329

[45] Leroux AE, Maugeri DA, Opperdoes FR, Cazzulo J, Nowicki C. Comparative studies on the biochemical properties of the malic enzymes from *Trypanosoma cruzi* and *Trypanosoma brucei*. FEMS Microbiology Letters, 2011; 314: 25–33. 10.1111/j.1574-6968.2010.02142.x 21105905

[46] Berná L, Chiribao ML, Greif G, Rodriguez M, Alvarez-Valin F, Robello C Transcriptomic analysis reveals metabolic switches and surface remodeling as key processes for stage transition in Trypanosoma cruzi. Peer J. 2017; 5: e3017 10.7717/peerj.3017 28286708 PMC5345387

[47] Liao Y, Smyth GK, Shi W. featureCounts: an efficient general purpose program for assigning sequence reads to genomic features. Bioinformatics. 2014 4 1;30(7):923–30. 10.1093/bioinformatics/btt656 24227677

[48] Feng J, Meyer CA, Wang Q, Liu JS, Shirley Liu X, Zhang Y. GFOLD: a generalized fold change for ranking differentially expressed genes from RNA-seq data. Bioinformatics. 2012; 28(21): 2782–2788. 10.1093/bioinformatics/bts515 22923299

[49] Aslett M, Aurrecoechea C, Berriman M, Brestelli J, Brunk BP, Carrington M, et al TriTrypDB: A functional genomic resource for the Trypanosomatidae. *Nucleic Acids Res* 38 (Database issue) 2009; D457–462. 10.1093/nar/gkp851 19843604 PMC2808979

[50] Martins C, Baptista CS, Ienne S, Cerqueira GC, Bartholomeu DC, Zingales B. Genomic organization and transcription analysis of the 195-bp satellite DNA in *Trypanosoma cruzi*. Mol Biochem Parasitol. 2008; 160(1): 60–4. 10.1016/j.molbiopara.2008.03.004 18440654

[51] Livak KJ, Schmittgen TD Analysis of relative gene expression data using real-time quantitative PCR and the 2(2Delta Delta C(T)) Method. Methods. 2001; 25: 402–408. 10.1006/meth.2001.1262 11846609

